# Curved Structure of Si by Improving Etching Direction Controllability in Magnetically Guided Metal-Assisted Chemical Etching

**DOI:** 10.3390/mi11080744

**Published:** 2020-07-30

**Authors:** Tae Kyoung Kim, Jee-Hwan Bae, Juyoung Kim, Min Kyung Cho, Yu-Chan Kim, Sungho Jin, Dongwon Chun

**Affiliations:** 1 Materials Science and Engineering, University of California at San Diego, 9500 Gilman Dr, La Jolla, CA 92093, USA; tkkim@a123systems.com (T.K.K.); jin1234jin@gamil.com (S.J.); 2 Advanced Analysis Center, Korea Institute of Science and Technology (KIST), Seoul 02792, Korea; jhbae@kist.re.kr (J.-H.B.); jykim1299@kist.re.kr (J.K.); 023381@kist.re.kr (M.K.C.); 3 Center for Biomaterials, Biomedical Research Institute, Korea Institute of Science and Technology (KIST), Seoul 02792, Korea; chany@kist.re.kr

**Keywords:** magnetically guided metal-assisted chemical etching, bulk Si etching, curved Si structure, catalyst encapsulation

## Abstract

Metal-assisted chemical etching (MACE) is widely used to fabricate micro-/nano-structured Si owing to its simplicity and cost-effectiveness. The technique of magnetically guided MACE, involving MACE with a tri-layer metal catalyst, was developed to improve etching speed as well as to adjust the etching direction using an external magnetic field. However, the controllability of the etching direction diminishes with an increase in the etching dimension, owing to the corrosion of Fe due to the etching solution; this impedes the wider application of this approach for the fabrication of complex micro Si structures. In this study, we modified a tri-layer metal catalyst (Au/Fe/Au), wherein the Fe layer was encapsulated to improve direction controllability; this improved controllability was achieved by protecting Fe against the corrosion caused by the etching solution. We demonstrated curved Si microgroove arrays via magnetically guided MACE with Fe encapsulated in the tri-layer catalyst. Furthermore, the curvature in the curved Si microarrays could be modulated via an external magnetic field, indicating that direction controllability could be maintained even for the magnetically guided MACE of bulk Si. The proposed fabrication method developed for producing curved Si microgroove arrays can be applied to electronic devices and micro-electromechanical systems.

## 1. Introduction

Fabrication techniques for micro/nano-structured Si, such as cantilever beams and bridge and buried microchannels fabricated using micro-electromechanical systems (MEMS) or nano-electromechanical systems (NEMS), have been intensively employed for a wide range of applications, including bio/chemical sensors [1,2], resonators [3], and power generators [4].

Dry-etching methods such as reactive ion etching (RIE) and deep reactive ion etching are important technologies for the micro/nano structuring of Si [5,6,7]. Recently, the Bosch process, which is a type of RIE, has been applied for the bulk etching of Si with a high aspect ratio, because it enables a high Si etching rate [8]. However, rough scallops on the surface and ion-induced defects impede the wider applications of this technique [9,10]. The deposition of C_4_F_8_, due to the passivation of the Si sidewall, is unfavorable when reactions are initiated at the Si surface, such as in battery anodes [10,11,12,13]. In addition, achieving control over the etching direction in dry-etching methods is difficult, thereby hindering the application of these methods for the fabrication of complex Si structures so that a combined lithography process is required to fabricate MEMS/NEMS structures. An alternative approach is to employ wet Si etching methods using KOH or TMAH (tetramethylammonium hydroxide) etchants to fabricate micro/nano-structured Si; however, the etching direction in such approaches is significantly restricted by the crystalline orientation of Si substrates [14,15,16].

By contrast, metal-assisted chemical etching (MACE) of Si using noble metal catalysts has garnered substantial attention in the past decade for fabricating micro/nano structures because of its simplicity, low cost, and ability to fabricate high-aspect-ratio structures such as pillar and hole arrays without sidewall etching [17,18,19,20,21]. In general, noble metal catalyst such as Pt, Ag, and Au are used in MACE to significantly improve the etching rate. This is because the Si etching rate in an etchant solution without a catalyst is considerably low because of the low catalytic ability of the Si surface [22]; therefore, only the Si underneath the metal catalyst undergoes considerably etching. Hence, when noble metal catalysts are patterned on the Si surface and immersed in a solution of HF and H_2_O_2_ diluted in DI water, a faster Si etching rate can be obtained using MACE, as compared to the use of Si without a noble metal catalyst. This implies that the geometry of patterned Si can readily be controlled by the size of the noble metal catalyst, etching time, and etchant concentration in MACE. The MACE of Si has been to fabricate various Si structures such as nanowires and microstructures [23,24,25,26,27,28,29].

Ideally, MEMS structures such as Si cantilever and bridge arrays can be obtained via a simple MACE procedure, provided the etching direction can be easily manipulated during the etching process. However, similar to dry/wet etching methods, controlling the etching direction during MACE is difficult, as the etching direction can only be modulated via the crystalline orientation of the Si substrate [30,31,32] and the concentration of the etchant [33]. To fabricate complex Si structures such as cantilevers and bridges, a type of MEMS structure, the etching direction should be sequentially altered during the etching process. However, the limited control over the etching direction hinders the direct fabrication of the MEMS structure via the MACE of Si. Therefore, a versatile technique for achieving etching direction controllability during MACE is necessary to enable the fabrication of various Si structures that are in demand for industrial applications.

To improve the etching performance in terms of speed and direction control, we developed a magnetically guided MACE process utilizing a magnetic Fe layer encapsulated in a multi-layered metal catalyst [34,35,36]. Herein, we used a conventional Au/Fe/Au multi-layer metal catalyst instead of a single noble metal; this enabled the adjustment of the etching direction owing to the magnetic pulling force between Fe and an external hard magnet placed underneath the etching bath. The curved Si structures were fabricated by changing the external magnetic field direction during the MACE of Si, the pattern size of which was less than 1 μm [34,36]. Uniform curved Si structures were fabricated via this method; however, as the pattern size in this case exceeded 1 μm, non-uniform curved Si structures were fabricated owing to an inhomogeneous magnetic pulling force, which resulted from the corrosion of Fe due to HF [36]. In addition, the usage of Au-coated magnetic nanoparticles for the curved nanostructure during the magnetically guided MACE of Si is unfavorable if the pattern size needs to be manipulated on a larger scale [34]. Consequently, an appropriate method to suppress the corrosion of Fe due to HF is essential for the fabrication of curved and zig/zag Si microstructures with a massive production yield. This would also eventually facilitate the eventual direct fabrication of MEMS structures such as cantilevers and bridges via MACE.

In this study, we design a novel tri-layer metal catalyst, wherein Fe is encapsulated by Au to suppress its removal by HF, thereby producing a uniform magnetic pulling force. As a result, uniform curved Si structures could be obtained in a bulk scale via magnetically guided MACE. We demonstrate that the tri-layer metal catalyst with encapsulated Fe can suppress Fe corrosion, which, in turn, enables the production of uniformly curved Si structures, as compared to those achieved using a conventional tri-layer metal catalyst.

## 2. Experimental

**Figure 1:**
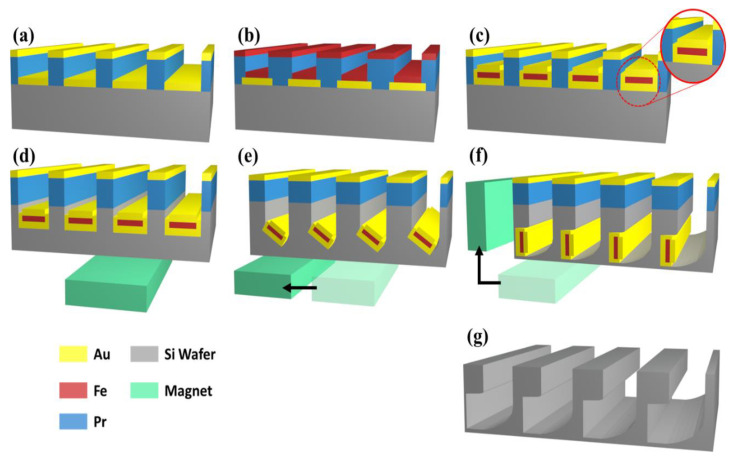
Fabrication process for magnetically guided metal-assisted chemical etching (MACE) of Si: (**a**) photoresist (PR) patterning and bottom Au catalyst (20 nm thickness) deposition, (**b**) PR patterning and magnetic Fe layer (10 nm thickness deposition), (**c**) PR patterning and Au (20 nm thickness) deposition for Fe encapsulation, (**d**), (**e**) and (**f**) magnetically guided MACE of by changing permanent magnet position, (**g**) Si groove arrays after the removal of Au and PR by chemical etching.

Figure 1 summarizes the process of fabricating curved Si structures via magnetically guided MACE. A 500-μm-thick p-type Si (100) wafer (boron-doped; resistivity of 10–20 Ω⋅cm) was utilized in this study. The Si chip size used for the experiment was 2 × 2 cm^2^. The Si wafer was pre-cleaned in acetone and ethanol for 10 min each. To encapsulate the Fe in the tri-layer Au/Fe/Au catalyst, a photoresist (PR; S1827, MNX, Long Beach, CA, USA; approximately 2.5 μm) line patterning was produced on the Si wafer surface via photolithography (Karl Suss; MA6, SUSS MicroTec, Garching, Germany), as illustrated in Section 3. Subsequently, the Au catalyst layer (20 nm) was deposited on the PR grooves via e-beam evaporation (Temescal BJD 1800 E-beam evaporator, Ferrotec, Santa Clara, CA, USA)). After the PR was removed, the line patterning of Fe was performed to encapsulate Fe with Au, in which the patterning width was narrower than the lower Au layer. Thereafter, the Fe layer (10 nm) was deposited via e-beam evaporation, followed by the removal of the PR. Finally, the PR was patterned such that the pattern width was identical to the lower Au layer. This was also followed by Au deposition (20 nm) via e-beam evaporation to ensure that the Fe layer was protected by the etching solution during the magnetically guide MACE of Si.

After the fabrication of the Au/Fe/Au catalyst encapsulated in Fe via the deposition of an upper Au layer, a post annealing process was performed at 150 °C for 5 h to improve etching performance [37]. High-temperature annealing processes can enhance the ferromagnetic properties of the Fe layer, improving the etching speed and the controllability of the etching direction by increasing the magnetic pulling force. Furthermore, the porous surface structure of the catalyst can be produced via an annealing process that facilitates the diffusion of etchant through the metal catalyst.

A mixture of diluted hydrofluoric acid (2 M) and hydrogen peroxide (9 M) diluted in deionized water was used as an etchant. The magnetically guided MACE of Si was conducted in a Teflon bath; a neodymium iron boron (NdFeB) permanent magnet (4 cm × 4 cm × 1 cm height) with an energy product strength of 35 MGOe was placed outside and underneath this bath to adjust the etching direction during the process. Si chips with the patterned tri-layer Au/Fe/Au catalyst encapsulated in Fe were immersed in the etchant for the magnetically guided MACE. The Si covered by the catalyst was etched faster than that covered by the PR, which can be explained as follows [38].

The reduction of hydrogen peroxide (H_2_O_2_) on the noble metal catalyst surface generates holes (h^+^) as follows:

(1) $$H_{2}O_{2}+2H^{+}\to 2H_{2}O+2h^{+}$$

Noble metal catalysts such as Au, Ag, and Pt facilitate the reduction of H_2_O_2_, thereby improving the etching speed by providing sufficient h^+^ than the pure Si surface. The holes generated near the noble metal surface pass through the noble metal surface and are injected into the valence band of Si, because the valance band potential of Si is lower than the redox potential between H_2_O and H_2_O_2_ [22]. This causes the dissolution of Si with HF, as follows:

(2) $$\mathrm{Si}+4h^{+}+6\mathrm{HF}\to {\mathrm{SiF}}_{6}^{2-}+6H^{+}$$

During the magnetically guided MACE of Si, the permanent magnet was shifted manually to alter the orientation of the magnetic field, which, in turn, changed the etching direction. The Si surface covered by the Au catalyst was etched along the direction of the external magnetic field because of both the enhanced etching rate through the catalyst reaction and the magnetic pulling force between the patterned ferromagnetic Fe and the hard magnet; the area covered by PR was protected because of its resistance to the etchant. The magnetically guided MACE of Si was performed at room temperature; after the etching process was completed, the sample was immersed in acetone and gold etchant to remove the remaining PR and metal catalyst. The etched Si chips were then washed with deionized water. Subsequently, the structure of the tri-layer metal catalyst of Au/Fe/Au and the etched Si structure were characterized using scanning electron microscopy (SEM; Regulus8230, Hitachi, Tokyo, Japan; Phillips XL30 ESEM, FEI, Hillsboro, OR, USA)). In addition, the surface morphology of the tri-layer metal catalyst was characterized via atomic force microscopy (AFM; Park XE7, Park Systems, Suwon, South Korea).

## 3. Results and Discussion

**Figure 2:**
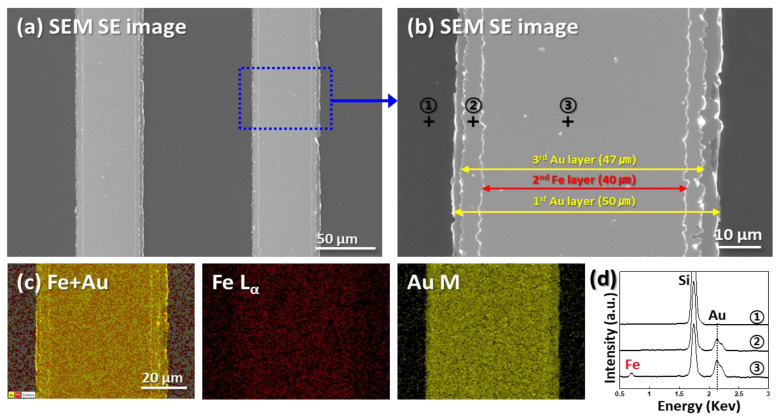
Scanning electron microscopy (SEM) images and energy-dispersive spectroscopy (EDS) images and spectrums of the tri-layer Au/Fe/Au metal catalyst encapsulating Fe after the annealing process.

Figure 2 presents the top-view SEM and energy-dispersive spectroscopy (EDS) images of the tri-layer metal catalyst with encapsulated Fe after the annealing process. Figure 2a,b show the tri-layer metal catalyst with Au (50-μm wide)/Fe (40-μm wide)/Au (47-μm wide) line patterns with 100-μm spacing fabricated on the Si surface via photolithography. This was confirmed by the EDS images of Fe and Au, as shown in Figure 2c; a 45-μm wide Fe layer is patterned between the Au layers. The EDS spectrum at point #2 exhibits sharp peaks at 2.123 KeV and 1.740 KeV corresponding to Au (M_α1_) and Si (K_α1_), respectively, whereas only an Si peak was detected at the EDS spectrum of #1, indicating that the lower (50 μm) and upper (47 μm) Au patterns were fabricated successfully. The width of the upper Au layer is narrower than that of the lower Au layer; this discrepancy might be due to a fabrication error. For the EDS spectrum at point #3, Fe L_α1_ peaks at 0.705 KeV corresponding to Au and Si (Figure 2d) were observed. This indicates that the tri-layer metal catalyst with encapsulated Fe was fabricated successfully.

**Figure 3:**
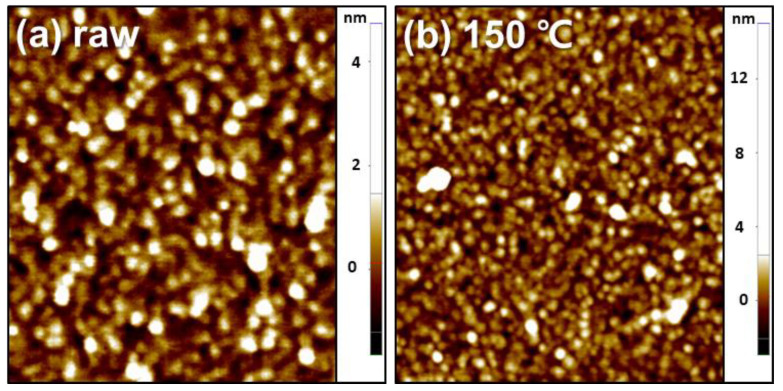
Atomic force microscopy (AFM) images of the tri-layer Au/Fe/Au metal catalyst with Fe encapsulation before (**a**) and after (**b**) the annealing process.

As the diffusion of the etchant depends on the surface morphology of the metal catalyst [21], porosity is an important factor influencing the etching speed. It has also been reported that enhanced porosity in the metal catalyst results in a higher etching speed [36]. Thus, to improve the porosity of the metal catalyst, we employed an annealing process after the formation of the tri-layer metal catalyst with Fe encapsulation. However, the changes in the surface morphology of the catalyst caused by annealing could not be clearly observed due to limitations of the image resolution of SEM. Hence, AFM was employed to analyze the surface morphologies before (Figure 3a) and after (Figure 3b) the annealing process. The root-mean-square roughness of the annealed metal catalyst was 1.038 nm (Figure 3b), whereas that of the as-deposited metal catalyst was 0.532 nm; this indicates that annealing provides a rough surface morphology that favors a higher etching during the magnetically guided MACE of Si, due to the shorter diffusion length of the etching solution [21].

**Figure 4:**
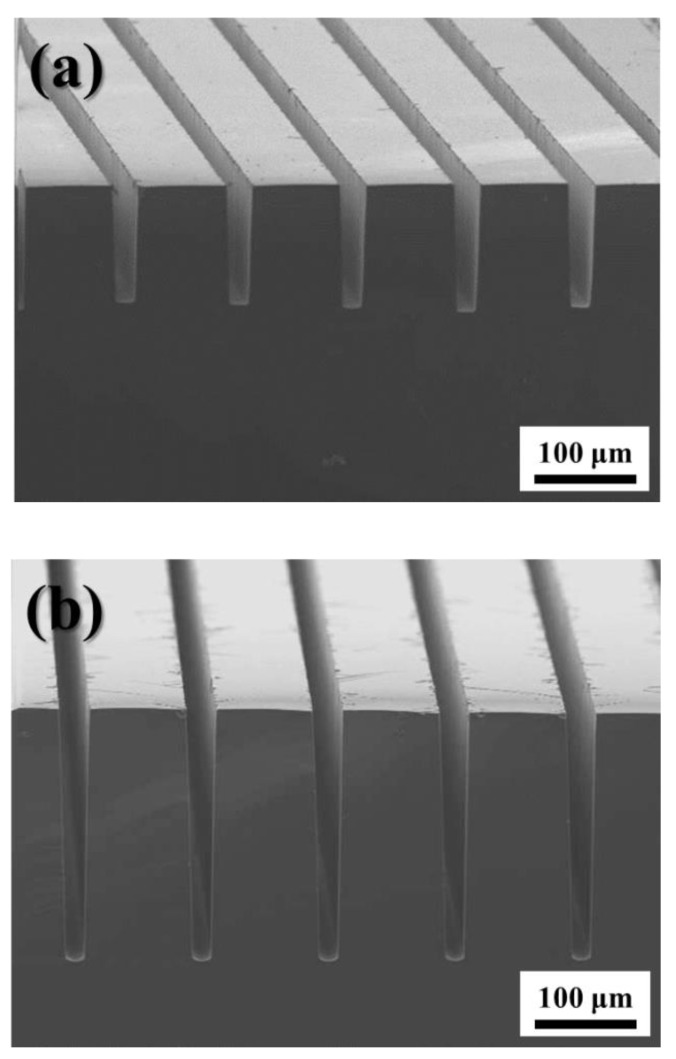
Cross-sectional SEM images of vertical Si groove arrays produced by magnetically guided MACE for etching durations of (**a**) 3 h and (**b**) 5 h.

**Figure 5:**
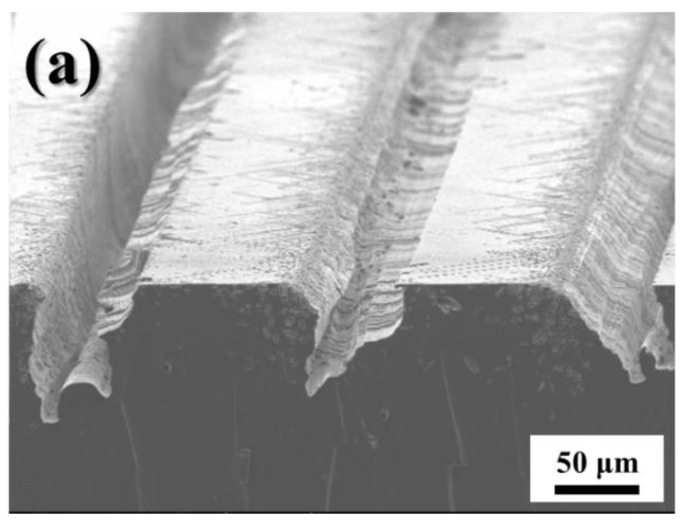

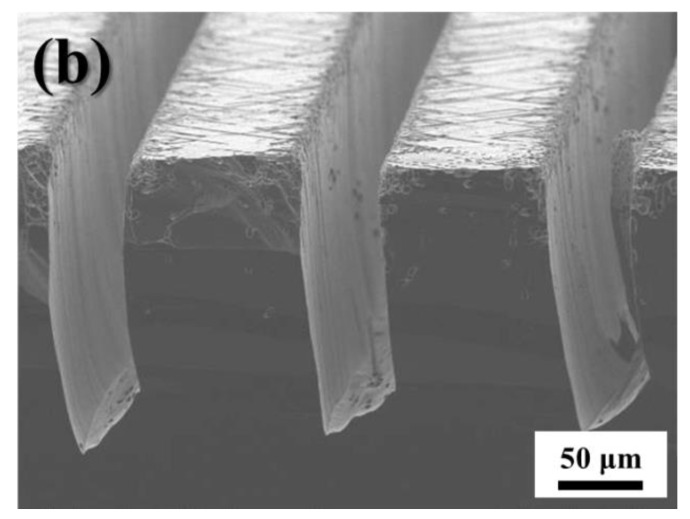
Curved Si groove arrays produced by magnetically guided MACE for 5 h using (**a**) a Swiss-cheese-like Au/Fe/Au catalyst and (**b**) Au/Fe_encapsulated_/Au catalyst (tilting angle: 30°).

Figure 4 shows the cross-sectional SEM images of vertical groove arrays produced via the magnetically guided MACE of Si for 3 h (Figure 4a) and 5 h (Figure 4b). The tri-layer metal catalyst of Au (20-μm wide)/Fe (15-μm wide)/Au (20-μm wide) lines spaced at 100 μm intervals were patterned on the surface of Si encapsulated with Fe, and the MACE of Si was performed under the magnetic field. During the etching, the strong magnet was placed underneath the Teflon beaker, and its position remained fixed for vertical etching.

Figure 4 confirms that the Au/Fe/Au tri-layer metal catalyst with encapsulated Fe is suitable for the magnetically guided MACE of Si in the vertical direction. The measured groove thickness for the etching of 3 h was 150 μm, while that for the etching of 5 h was 240 μm; these correspond to etching speeds of 0.83 μm/min (Figure 4a) and 0.8 μm/min (Figure 4b), respectively. Likewise, a reduction in the etching speed with etching time was observed in our previous results, which were obtained using a conventional Au/Fe/Au tri-layer metal catalyst [34,35]. A direct comparison between etching speed and catalyst structure is difficult because etching speed depends on not only the catalyst structure but etchant concentration and the size of the catalyst patterned on the Si surface during magnetically guided MACE. However, the reduction in etching speed with increasing time appears to be mitigated by the encapsulation of Fe, as compared to the etching speed for a conventional Au/Fe/Au catalyst [34,35]. This reduction in etching speed with the increase in etching time can be attributed to the reduction in the volume of Fe caused by the HF-induced corrosion, which decreases the magnetic pulling force between Fe and the permanent magnet. Unlike the conventional Au/Fe/Au tri-layer catalyst, the sidewalls of the Fe layer in our catalyst remain protected due to the encapsulation by Au; this effectively suppresses Fe corrosion. As a result, the reduction in etching speed with an increase in etching time is not appreciable during the magnetically guided MACE of Si. It should be noted that a slightly tapered structure was produced. The loss at both Au layers in the metal catalyst contribute toward the corrosion of Fe, resulting in a reduction of catalyst dimensions with increasing etching time; this could potentially explain the tapered Si structure obtained. Moreover, this indicates that the corrosion of Fe could not be prevented completely, even when Fe is encapsulated by Au.

Figure 5 shows the Si groove arrays produced by magnetically guided MACE for 5 h using a conventional tri-layer catalyst (Figure 5a) and the tri-layer catalyst with encapsulated Fe (Figure 5b). The tri-layer metal catalyst of Au (50 μm wide)/Fe (40 μm wide)/Au (50 μm wide) line patterns were produced on the surface of Si encapsulated by Fe. To adjust the etching direction for fabricating a curved Si structure via magnetically guided MACE, the magnet underneath the beaker was moved gradually from a vertical position to a tilting angle of 30° during the etching.

**Figure 6:**
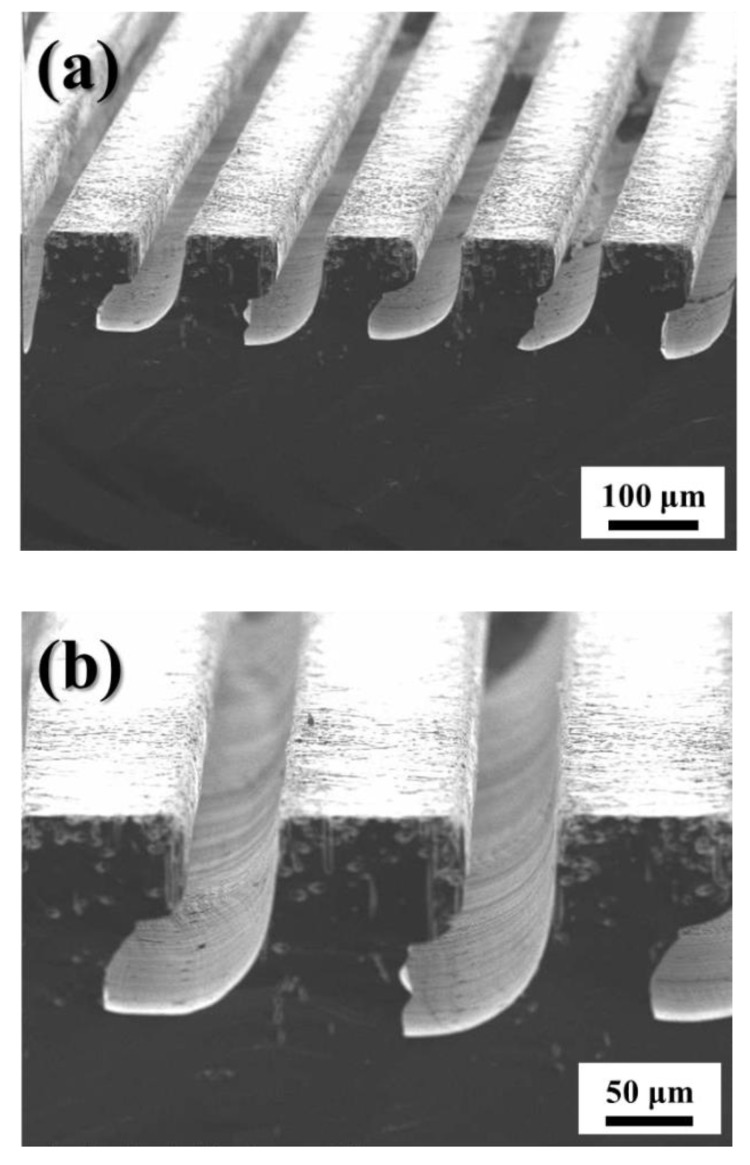
Curved Si groove arrays produced by magnetically guided MACE for 5 h using (**a**) a Swiss-cheese-like Au/Fe/Au catalyst and (**b**) Au / Fe_encapsulated_ /Au catalyst (tilting angle: 90°).

As shown in Figure 5a, 60-μm-thick and non-uniform Si grooves with rough surfaces were produced by etching with a conventional tri-layer catalyst, although rougher surface morphology was produced by annealing process, as shown in Figure 3, which reduces the diffusion length of the etchant. Changing the etching direction results in an unstable Si/catalyst interface, thereby facilitating Fe corrosion because the etchant can readily penetrate the interface and etch the Fe. Therefore, as the Fe is not sufficiently protected given the changing direction of the magnetic field, magnetically guided MACE of Si is infeasible due to the reduced and inhomogeneous magnetic pulling force. In contrast, uniform 150-μm-wide curved Si groove arrays with smooth surfaces were produced during the magnetically guided MACE of Si using the tri-layer metal catalyst with encapsulated Fe, as shown in Figure 6b; this indicates that the etching direction can be effectively controlled by changing the direction of the magnetic field when the catalyst includes encapsulated Fe, thereby proving that the HF-induced corrosion of Fe can be effectively suppressed via encapsulation.

The etching speeds calculated from the thickness of the measured grooves in Figure 5a and Figure 4b are approximately 2 μm/min and 0.5 μm/min, respectively. Fe corrosion could result in partial Au ejection because of the strong adhesive force between Fe/Au, as compared with that for Au/Si. This would result in a lower etching speed and a rougher surface when using magnetically guided MACE of Si with a conventional tri-layer metal catalyst, as shown in Figure 6a. In contrast, a higher etching speed and a smooth surface morphology were obtained when the tri-layer metal catalyst with encapsulated Fe was used, indicating the partial Au ejection was successfully prevented by the suppression of Fe corrosion.

Figure 6 presents the curved Si groove arrays produced by magnetically guided MACE for 5 h using the tri-layer metal catalyst with encapsulated Fe. The tri-layer metal catalyst of Au (50-μm wide)/Fe (40-μm wide)/Au (50-μm wide) line patterns spaced at 100 μm intervals were fabricated on the surface of Si. First, the magnetically guided MACE of Si was performed by using a strong magnet placed vertically under a beaker. After this vertical etching for 1 h, the position of the hard magnet was gradually changed to a 90° tilting angle, and etching was conducted for 2 h under a lateral magnetic field.

As shown in Figure 6, a curved Si structure with a smooth surface was produced via magnetically guided MACE when the tri-layer metal catalyst with encapsulated Fe was used. As expected, the curvature of the Si structure was enhanced on increasing the tilting angle from 30° to 90°, indicating that the etching direction could be adjusted effectively by using the external magnetic field.

Finally, the bulk micromachining of Si with a curved structure was successful conducted via the magnetically guided MACE of Si using the metal catalyst with Fe encapsulated in an Au (50 μm wide)/Fe (40 μm wide)/Au (50 μm wide) tri-layer. Therefore, it is evident that suppressing the corrosion of Fe, through Fe encapsulation, improves the controllability of etching direction during the magnetically guided MACE of Si.

## 4. Conclusions

Uniform curved Si groove arrays were produced using encapsulated Fe in a tri-layer metal catalyst (Au/Fe/Au); this catalyst improved the controllability of etching direction during magnetically guided MACE. A magnetic layer in a tri-layer metal catalyst was used to modulate the etching direction via a magnetic pulling force. To improve the direction controllability during magnetically guided MACE, the Fe layer was encapsulated with Au, which suppresses the HF-induced corrosion of Fe, thereby enabling the production of a uniform magnetic pulling force.

Uniform curved Si groove arrays were produced via magnetically guided MACE using this Fe encapsulation, whereby the etching direction was manipulated by moving the external strong magnet during this process. By contrast, non-uniform Si groove arrays with rougher surfaces were obtained when using a conventional tri-layer metal catalyst. This indicates that the encapsulation of Fe with Au can effectively protect Fe against corrosion due to HF, resulting in an enhanced direction controllability. Furthermore, it was observed that the curvature of Si groove arrays can be modulated by adjusting the tilting angle of the external strong magnet, when using encapsulated Fe in the tri-layer metal catalyst.

We also attempted to fabricate complex Si structures such as Si cantilever arrays and zig-zag microwire arrays; however, this could not be achieved using the proposed approach. On increasing the etching time, the controllability of etching direction decreases, which is likely caused by the corrosion of Fe due to HF. In addition, depletion of the etchant around etched areas between the metal catalyst and Si could result in a reduced etching rate as well as poor controllability over etching direction. To directly fabricate MEMS structures via the magnetically guided MACE of Si, magnetic alloy materials such as AuFe and CoFe need to be used as the metal catalyst in order to reduce the corrosion of the magnetic layer by HF. In addition, the etching process should be conducted under an etchant circulation system to ensure sufficient etchant reaches the reactive area which remain as future works. Although complex Si structures could not be achieved via magnetically guided MACE with Fe encapsulation, the results of this study prove that encapsulation helps prevent Fe corrosion, resulting in a uniform magnetic pulling force. Consequently, uniform curved Si structures can be fabricated in bulk by improving the controllability of etching direction during magnetically guided MACE.
